# Rare blood group registry in India-current challenges and future perspectives

**DOI:** 10.3389/fgene.2023.1264853

**Published:** 2023-09-11

**Authors:** Suvro Sankha Datta, Suhasini Sil, Saikat Mandal

**Affiliations:** ^1^ Department of Transfusion Medicine, Tata Medical Center, Kolkata, India; ^2^ Department of Transfusion Medicine, All India Institute of Medical Sciences, New Delhi, India; ^3^ Young Professional Council, International Society of Blood Transfusion, Amsterdam, Netherlands; ^4^ Medical Oncology, Hull York Medical School, Hull, United Kingdom

**Keywords:** rare blood groups, registry, immunohematological approach, blood group genotyping, India

## Abstract

Patients who require blood from rare blood group donors present great challenges even to the most advanced healthcare delivery system. It is most challenging to supply blood for a patient with an antibody to an antigen of high prevalence. The blood donor lacking the corresponding antigen would have an occurrence rate of less than one in 1,000. The International Rare Donor Panel was established in 1965, but since then there has been gross underrepresentation of South Asian countries, including India. There are several challenges to starting a rare blood group donor program in India that include technical, logistical, and administrative limitations. But the main limiting factors are poor availability of trained resources, lack of awareness, absence of antibody screening, inadequate number of laboratories with blood group genotyping facilities, and the decentralized nature of blood transfusion services. Despite that, there were several rare blood groups identified by Indian immunohematologists in the recent past. Recently, a transfusion genomic group has been established in collaboration with the clinical transfusion medicine specialists in India under the GUaRDIAN (Genomics for Understanding Rare Disease in India Alliance Network) initiative to address the domain of rare blood group genomics. Similarly, the National Institute of Immunohematology, Mumbai under the directive of the ICMR (Indian Council of Medical Research) has taken a step to start the RDRI (Rare Donor Registry of India). In this context, we explore the current challenges of setting-up a rare blood group registry in India and future goals from a developing nation’s perspective.

## 1 Introduction

The International Rare Donor Panel (IRDP) was established in 1965 under the directive of the International Society of Blood Transfusion (ISBT). It was one of the earliest initiatives of international collaboration with the World Health Organization (WHO). The compilation and maintenance of the IRDP has been assigned to the red cell reference department of the International Blood Group Reference Laboratory (IBGRL) since that time (Thornton, 2016). However, The American Rare Donor Program (ARDP) was formed in 1998 as a collaborative effort between the AABB (formerly called the American Association of Blood Banks) and the American Red Cross to provide rare blood for patients in need. The ARDP database, REGGI, is maintained at the Philadelphia ARDP site (Flickinger, 2014). Patients who require blood from rare blood group donors present great challenges even to the most advanced healthcare delivery system. It requires a massive effort to supply blood for a patient with an antibody to an antigen of high prevalence or antibodies against multiple common erythrocyte antigens. Among the Asian countries, Japan, China, Taiwan, Israel, Singapore and Iran, are currently enrolled in IRDP. Therefore, a significant proportion of the global population who are living in South Asia, including in the Indian subcontinent, does not have any access to such a facility. There are several technical, logistical, and administrative challenges to starting a rare blood group donor program in India. Starting from the ethnic and racial diversity of the inhabitants to the cost of the rare antisera, the list is never ending. In the current article, we critically explore the current challenges of setting-up a rare blood group registry in India and the way forward from a developing nation’s perspective.

### 1.1 Need of a rare blood donor registry in India

A rare blood donor phenotype occurs in 1 in 1,000 donors that includes high-prevalence antigen negative or multiple common antigen negative blood groups (Flickinger, 2014; Thornton, 2016). The rare blood group phenotypes encountered in each country may vary according to the differences in ethnicity. Over a period of time, Bombay/para-Bombay,—D −/− D -, In (a+b-), Co (a-b-), I-i-, CdE/CdE, Mg, P-null and Emm are some of the rare blood group phenotypes that are reported in India (Joshi and Vasantha, 2012; Shastry et al., 2020; Shah et al., 2021). Bombay phenotype is the most requested rare blood group in India (Talukder et al., 2014). A Mumbai-based survey showed that the incidence is 1: 7,600 in Mumbai city to 1: 2,500 in certain parts of Maharashtra (Agarwal et al., 2016). In India, the medical need accounted for 41.8% of blood requests followed by surgery (25.3%), pediatrics (19.0%), and obstetrics (13.8%) (Mammen et al., 2021). But clinical guidelines often overlook the critical aspects of managing patients with unique blood groups. Each blood center strives hard to provide blood in a timely manner for patients in need, but it is extremely difficult to obtain compatible units for those with rare phenotypes in the absence of a structured national rare blood donor registry. Considering the magnitude of the problem, a robust program is required to meet the rare blood needs of the country.

## 2 Lack of genotyping/phenotyping facilities

There are a few key factors for rare donor identification. First, a sustainable cost-effective programme of red cell antigen typing in large cohorts of donors using a high-throughput technique is required. A programme of mass antigen screening is essential to provide information about the phenotypes of the donor population and secure the ability to maintain a stable and sufficient daily inventory of antigen negative red cell units (Paccapelo, 2018). Second, donors with a rare blood group should be suspected when a routine antibody screening test is positive for antibodies to high-prevalence antigens. Third, individuals with a rare blood group are mostly identified during the pre-transfusion testing or antibody screening during the antenatal period. All patients who are eligible at a later date to be a potential blood donor should be included in the rare group registry and encouraged to donate for future needs. Lastly, donor screening of family members, especially siblings of patients sensitized to high-prevalence antigens, should be encouraged. The chance of getting a rare blood group is higher in this group than in the random population. The same is true for screening of donors from a similar racial or ethnic background, which could be of great value (Yang et al., 2022). But for all these activities, the primary requirement is to have access to the rare group antisera and blood group genotyping facility, which is still at a very nascent stage in India. In fact, the donor and patient antibody screening is still not being performed in most of the blood centres across the country due to the cost of the screening panels. Even if the antibody screening is performed on antenatal women, the testing is predominantly restricted to RhD-negative mothers and pooled red cells are used instead of screening panels, which is a far less sensitive way of testing. Considering all these technical challenges, there are multiple hurdles that need to be overcome before starting a rare group registry in India.

## 3 Challenges to form and maintain a rare blood group database

In most countries, one national or a few regional reference laboratories are responsible for the screening, selection and supply of rare blood. They perform regular typing of a large number of donors in different demographic locations to set up registries of rare donors. These centres have specific standard operating procedures (SOP) designed to support the activities related to the handling of advanced immunohematology procedures and rare blood units. It is a major challenge in a country like India with a decentralized blood transfusion service to form and maintain a unanimous database for rare group donors. Again, most of the blood donors are one-time donors in India and currently there is no such system in place to capture the relocation details of the regular voluntary donors in the country. Drafting a standard SOP for rare blood group identification and the use of comprehensive information technology to enter the donor details in a web-based database would be the first step that is required before starting a rare group registry in India. There has to be a validated system in place to update the donor demographic details, availability, and eligibility for donation at frequent intervals in this network.

## 4 Lack of freezing facility and maintenance of storage system

**FIGURE 1:**
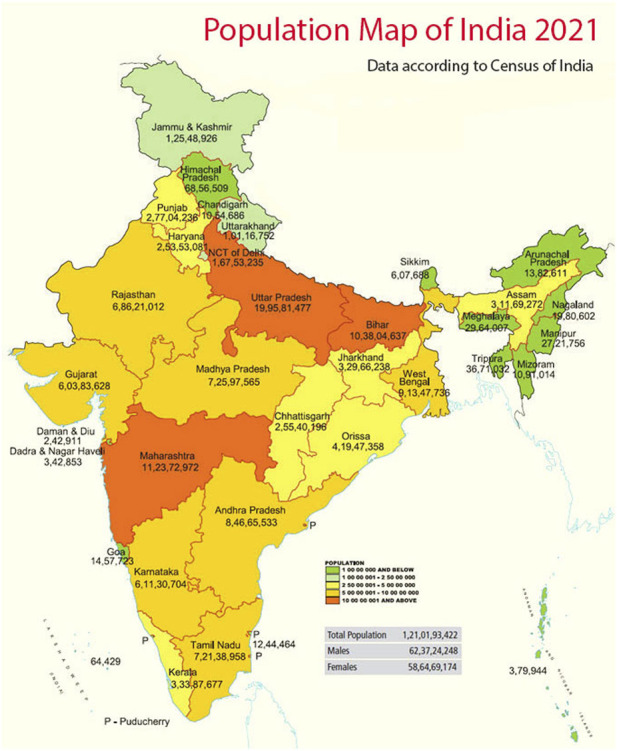
Population and provinces of India.

Glycerol is the most commonly used RBC cryoprotectant, and most RBC units, when frozen, are stored at −80°C (40% wt/vol glycerol, high-glycerol method). Frozen RBC usually expires after 10 years, but few countries permit storage of frozen units beyond 10 years for extremely rare groups. All frozen rare blood units must be thawed, and prior to transfusion, the glycerol must be removed. The use of thawed RBCs is limited to 24-h expiration when stored at 1°C–6°C and an open system is used, but they may be stored up to 14 days if a closed system (ACP 215 system, Haemonetics) is used for thawing (Meryman and Hornblower, 1977). Technical maintenance of such a storage system is very crucial and any change in temperature should be recorded vigilantly by the engineering staff in order to ensure proper operation of equipment. Risk mitigation strategies should be in place for emergency situations such as break-downs. The equipment should be well-equipped with an audible alarm enabling it to sense any abnormal situation and should have an alternative power supply source. Unfortunately, not even a single frozen red cell storage facility is operational in India. Also, there is no such standard SOP available for frozen red cells in the country. Merely identifying an individual with a rare blood group is of no use if even a single frozen inventory is unavailable in a country with an estimated population of 1.42 billion at present (Figure 1). Therefore, a frozen inventory of rare units is the need of the hour as it is blood that is immediately available to be used to respond to an urgent need rather than recruiting, collecting, and processing a rare donor.

## 5 Challenges related to transportation of a rare blood unit

A reliable transportation of the rare blood unit to the intended recipient is critical for safe blood transfusion. As the majority of transfusion reactions arise from transcription error, confirmation of serological antibody testing and cross-matching of any rare RBC units at source should be done before shipping them. In order to transport the rare blood unit, insulated and leak-proof boxes that are validated for 48-h transit time should be used. Low-temperature storage could be maintained either with wet ice for liquid units or dry ice for frozen units. For long distances, transport time can be extended and it is easier to ship frozen cells if available, but a deglycerolizing facility should be available at the receiving facility. Again, transportation in the liquid state is always preferred as the chance of breakage is much less. It is necessary to use a data logger to record temperature information during transportation. Labelling of containers is also very important. The labels should be clear and have the full shipper address, full details of properly classified biological or infectious material, and the information on the box should clearly indicate the contact person in case of any delay in transportation. Considering the significant distances between different parts of India and the conditions of roads, especially in remote areas, it would have been a substantial challenge to deliver a rare blood unit to the intended hospital where the patient is admitted in a timely manner to meet the clinical requirement.

## 6 Lack of awareness and knowledge

One of the key factors to managing the requirement of a rare blood unit is the knowledge and awareness of the doctors and technical staff working in the blood transfusion services. Clear communication between different blood centres is the utmost important, be it involved in requesting a rare blood unit for a patient or receiving a rare blood shipment. A nodal person has to be assigned to each blood center for communicating the relevant information, including the request for transfusion, the patient’s blood sample, consignments, etc. In India, compliance with proper communication and collaboration is still very little and that might be attributed to lack of overall awareness and knowledge.

## 7 Inadequate training in advanced immunohematology

Blood center staffs need to be trained in rare blood collection, processing, compatibility testing, storage, and distribution. These personnel are required to have a standard educational qualification, job training and experience to perform their duties. Continuous educational activities of the employees are critical for their training to perform advanced immunohematological tests. They should be offered proper remuneration based on their expertise to reduce the rate of attrition. Again, in India there is a significant lack of a trained workforce in blood transfusion services. This is not merely restricted to the technical staff. There are very few doctors available who are well trained in advanced immunohematology techniques. The physicians are not well oriented, either, because transfusion medicine is not taught as a separate subject at undergraduate level. Therefore, there is almost always chaos whenever any requirement arises for a patient with a rare blood group in India.

## 8 Financial constraints

There is a high cost involvement because of the equipment and consumables used for a rare blood group programme. Freezing and storage devices require substantial budgeting. Therefore, dedicated initiatives from the government are required to provide financial support to the blood centres. According to a survey, the cost of collecting, testing, storing and transporting blood to a blood centre in the United States is approximately $925 to $1,150 per RBC unit (Meny et al., 2013). According to the ARDP, the cost of special antigen typing has been raised to $500—$1,200 for each unit. Thus, the cost of a rare unit of blood in the US ranges from approximately $1,148 to $1,373, which is too much for a low- and middle-income country like India. Ultimately, neither the patient nor his insurer will be able to bear such a high cost of transfusion.

## 9 The way forward

**FIGURE 2:**
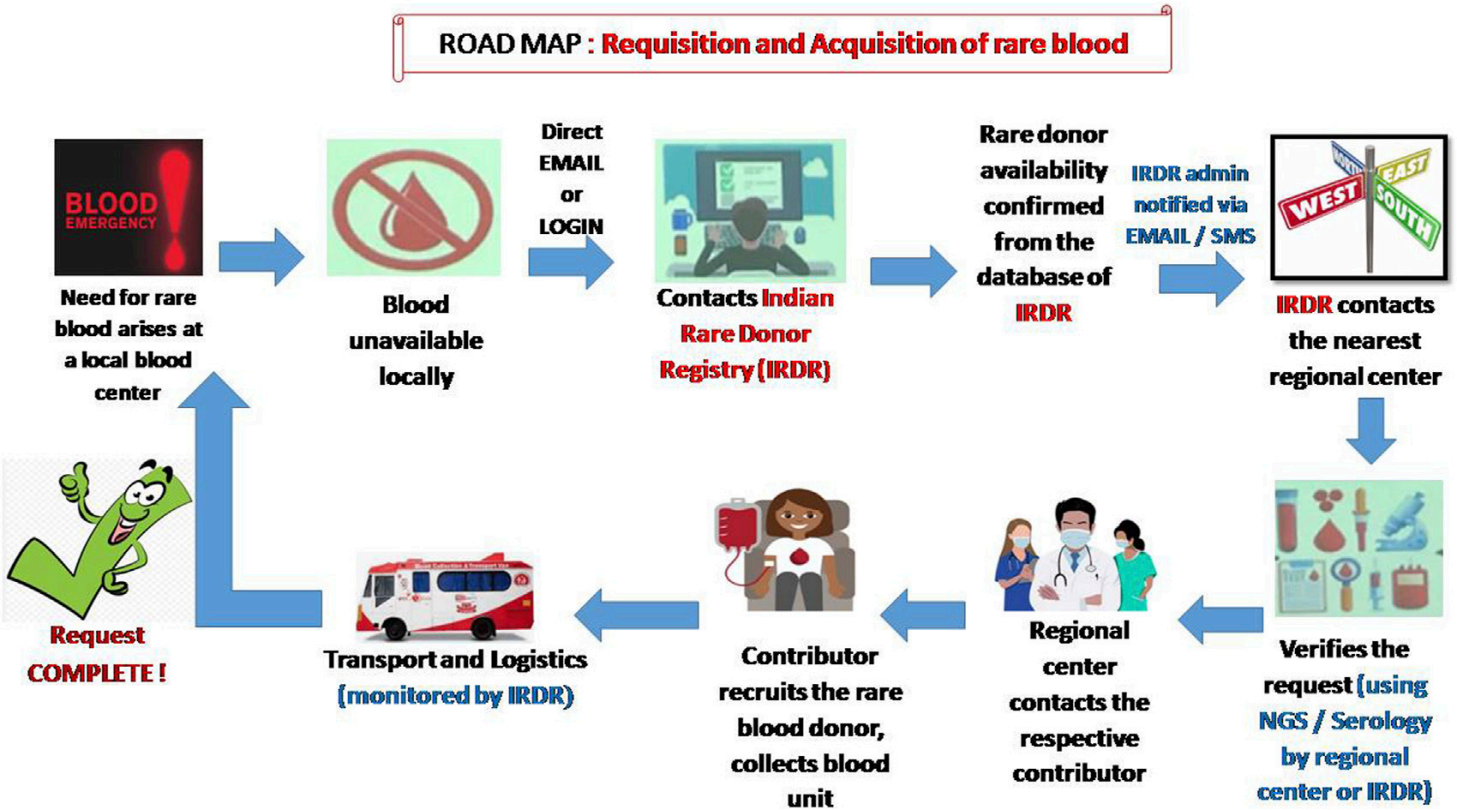
A possible road map to obtain a rare blood unit in India.

The screening to identify rare groups of individuals may be initiated in India by starting a national program of antibody screening for donors, patients and antenatal women. This may be done efficiently and cost-effectively on a large scale for all clinically significant blood groups using a high-throughput platform (Mathur et al., 2022). It is a well-known fact that mass-scale RBC genotyping is a major alternative to RBC phenotyping to increase inventories of antigen negative blood components, especially when serological reagents are unavailable (Denomme and Anani, 2019). However, in a country like India where money is the major obstacle to starting red cell genotyping, an alternative method such as multiplex polymerase chain reaction with DNA pooling might be used as a cost-effective strategy for genotyping rare blood types (He et al., 2013). Another simple method such as the urea method for screening for the Jk (a–b–) phenotype might be useful, as glycoproteins of the Kidd blood group system serve as a urea transport channel on the cell membrane of erythrocytes (Hamilton, 2015). Despite all the pre-analytical and analytical variables, monocyte monolayer assays (MMAs) can also be considered as an alternative in certain situations. The MMA assay uses antigen positive and antigen negative red cells that are sensitized to the patient’s serum. If the MMA test result indicates that the implicating antibody is not stimulating monocyte activity, there is a high likelihood that antigen-positive RBC products can be transfused safely to the recipient (Tong et al., 2019). Even if rare blood donors have been identified and recruited, they may no longer be active or traceable. For this reason, it is very important to stay in touch with donors and maintain regular contact. Distributing letters of appreciation or encouraging them by using different interactive social media could be an effective way of motivating them to donate again (Gammon et al., 2021). Once recruited, a wallet-sized card with the donor’s name and phenotype, address and the importance of the donor’s blood for rare patients should be given to the donor for future use (Laureano et al., 2023). Alternately, a dedicated National Rare Donor Day for rare donor recognition might be initiated in the country and efforts must be made to cover these activities by the national media (Moghaddam, 2016). To understand the current need for frozen red cell inventory, a simulation model should be adopted first. This could predict the optimal number of units that is required to be held in frozen inventory and their impact on the supply chain (Blake and Clarke, 2019). Once a request is received for rare blood, it is important to gather clinical information of the patient. The implementation of a patient blood management program is crucial at the hospital level to justify the need for transfusion (Meny et al., 2013). In some situations, the underlying cause of anemia can be corrected without transfusion support, or autologous donations can be considered in a patient undergoing elective surgery (Laureano et al., 2023). In Figure 2 we propose a possible road map to obtain a rare blood unit in India when a need arises at a local blood centre. In an effort to improve availability and delivery times of a rare blood unit, an unmanned aerial vehicle (drone) might be considered to deliver the blood products to remote healthcare facilities (Nisingizwe et al., 2022). Frequent training of technical and communication skills as well as proficiency testing of technical staff at a regular interval should be conducted in all blood centres. Dedicated staff should be identified first for undergoing the advanced immunohaematology training. The role of a well-developed regional or national immunohematology reference laboratory (IRL) is extremely crucial not only for the identification and confirmation of a rare blood group but also for providing requisite training to the staff of different blood centers across the country. Steps need to be taken to integrate transfusion medicine into medical curriculums at undergraduate level. This could improve the perceptions of physicians and strengthen the process of patient blood management.

## 10 Discussion

In summary, a rare donor program requires perseverance, patience, and long-term commitment, given that one cannot predict when rare blood will be needed. Although the need for rare blood is uncommon, when the need arises, it becomes a matter of life and death. During the past five decades, interest in the national rare donor program has increased in a significant manner worldwide. This is especially seen in high income countries. In low- and middle-income countries, however, the experience is not so good (Ghezelbash et al., 2018). In India, some efforts are being made at regional level (Setya et al., 2020; Polavarapu et al., 2021). Developing BGvar (Blood group associated genomic variant resource) is one such initiative (Rophina et al., 2022). Also, the recent identification of anti-Hro alloantibody in D--individuals is an example of how scientific collaboration can contribute to improving patient care (Chowdhry et al., 2019; Kulkarni et al., 2022). The e-Rakt Kosh network might be of use in future for capturing the rare donor data in India (BLDAHIMS, 2023). However, the National Institute of Immunohematology, Mumbai under the aegis of the ICMR has already registered the details of approximately 500 rare group donors in India along with their consent and it is expected to be increased by many folds after starting the RDRI. As the majority of the patients with multiple antibodies would have hemoglobinopathy and require life-long transfusion, therefore, it is essential to provide special blood group cards to these patients by clearly mentioning the complete phenotype and alloantibody status. In general, transfusion laboratories in India are not equipped with blood cells of all the different rare phenotypes (Reddy et al., 2023). Hence, efforts must be made to designate and manage different IRLs through collaboration between academia and the governments. The present migration of people throughout the world and increasing medical tourism in India may well result in further rare blood group related serological challenges. As the cost of genotyping is decreasing, therefore, this is likely to result in the implementation of molecular technology in different blood centers. Finally, a structural program should be in place that allows the need for blood at a local level to be disseminated to the national network. To conclude, financial and administrative support of the government, the educational program of technical officers and clinicians along with collaboration with other adjacent low-middle income countries is essential for establishing a successful and effective rare blood group registry in India.

## Data Availability

The original contributions presented in the study are included in the article/supplementary materials, further inquiries can be directed to the corresponding author.
